# Midterm Results of Severe Hip Dysplasia after Using a Cementless Acetabular Component with Bulk Bone Graft in Total Hip Arthroplasty: A Minimum Five-Year Follow-Up Study

**DOI:** 10.3390/bioengineering11080841

**Published:** 2024-08-19

**Authors:** Takuya Konno, Tomohiro Shimizu, Masahiro Inoue, Takeshi Masuda, Mohamad Alaa Terkawi, Norimasa Iwasaki, Daisuke Takahashi

**Affiliations:** 1 Department of Orthopaedic Surgery, Wajokai Eniwa Hospital, Eniwa 061-1449, Japan; t_konno9696@yahoo.co.jp (T.K.); ino0017@arion.ocn.ne.jp (M.I.); otayori@eniwa-hosp.com (T.M.); 2 Department of Orthopaedic Surgery, Faculty of Medicine and Graduate School of Medicine, Hokkaido University, Sapporo 060-8638, Japan; materkawi@gmail.com (M.A.T.); niwasaki@med.hokudai.ac.jp (N.I.)

**Keywords:** bulk bone autograft, total hip arthroplasty, cementless acetabular component, severe acetabular dysplasia

## Abstract

In patients with severe hip dysplasia, total hip arthroplasty (THA) using bulk bone graft (BBG) enhances anatomic cup positioning and provides early structural support. This study assesses the mid-term outcomes of THA with BBG in patients with over 50% graft bone coverage. Among 1951 patients who underwent THA between 2003 and 2007, 183 had BBG. After excluding early dropouts and infections, 151 patients remained. They were classified into uncovered (<50% coverage, 79 patients) and covered (>50% coverage, 72 patients) groups. The efficacy of cup fixation was compared between these groups. After ten years, the survival rate for not needing THA revision was 98% in the uncovered group and 100% in the covered group, while the rate for radiographic stability was 93% versus 99%, respectively. Although the cutoff value for the uncovered portion could not be clarified in this study, the mid-term results for 50% to approximately 70% uncovered were comparable to those for 50% or lesser, which have previously been expected to perform well. Recently, biomechanically advantageous bone grafting techniques have been identified, and based on the results of this study, it may be possible to expand the indications for THA with bone grafting for developmental dysplasia of the hip.

## 1. Introduction

The acetabulum often has an irregular shape in patients with developmental dysplasia of the hip (DDH), particularly in Crowe types II and III [1,2,3]. An obvious bone defect is frequently observed above the acetabular component after reconstruction at the true acetabulum level during total hip arthroplasty (THA). Large bone defects may affect the initial stability of the component and require the application of a structural bone graft [4]. Bone defects in the acetabular roof are thought to disrupt conventional methods of acetabular component implantation. Various reconstructive methods have been used to address these defects, including superior placement of the cup [5,6], medialization of the cup [7,8], and augmentation of bone stock with fixation of a bulk bone autograft on the anterolateral aspect of the acetabulum, followed by implantation of a cemented or cementless cup [9,10,11,12,13,14].

Bulk bone grafting (BBG) allows for a better anatomic cup position, provides early structural support for the acetabular component, and, if incorporated, offers beneficial bone stock for any future revision surgery. For these reasons, treatment with BBG is sometimes preferred in THA for patients with a large uncovered area above the cementless acetabular component [15,16]. Numerous studies have shown excellent results with a cementless acetabular component and a bulk bone autograft [15,16,17,18,19]. A recent study using finite element analysis has revealed biomechanical differences in bone grafting methods, such as BBG, and has proposed useful surgical techniques from a biomechanical perspective [20]. However, clinically, the amount of graft bone coverage required for secure fixation of the cementless acetabular component has not been definitively proven.

Earlier reports have demonstrated the outcome of treatment for graft bone coverage of ≥50% [9,10,21,22,23,24]. Therefore, the aim of this study was to compare the mid-term results of THA combined with a cementless acetabular component and a bulk bone autograft for acetabular reconstruction in dysplasia patients with an exceeding cementless acetabular component over 50%.

## 2. Materials and Methods

This retrospective study was conducted in accordance with the ethical standards of the Declaration of Helsinki and approved by the institutional review board (#015-0205). All patients were informed about the study and provided consent for its publication when they decided to undergo THA surgery. A total of 1951 patients received THA at the institution from January 2003 to November 2007. Of these, 183 patients were treated with THA using cementless acetabular component fixation to the original acetabulum and reconstruction of acetabular defects by grafting bulk bone from femoral heads, and they were included in this retrospective study. Of these, 31 cases were excluded due to failure to follow up over 5 years after surgery because of hospital changes and difficulty in going to the hospital, and 1 case was excluded due to early revision surgery for infection (resulting in a 5-year follow-up ratio of 82.5%).

**Figure 1:**
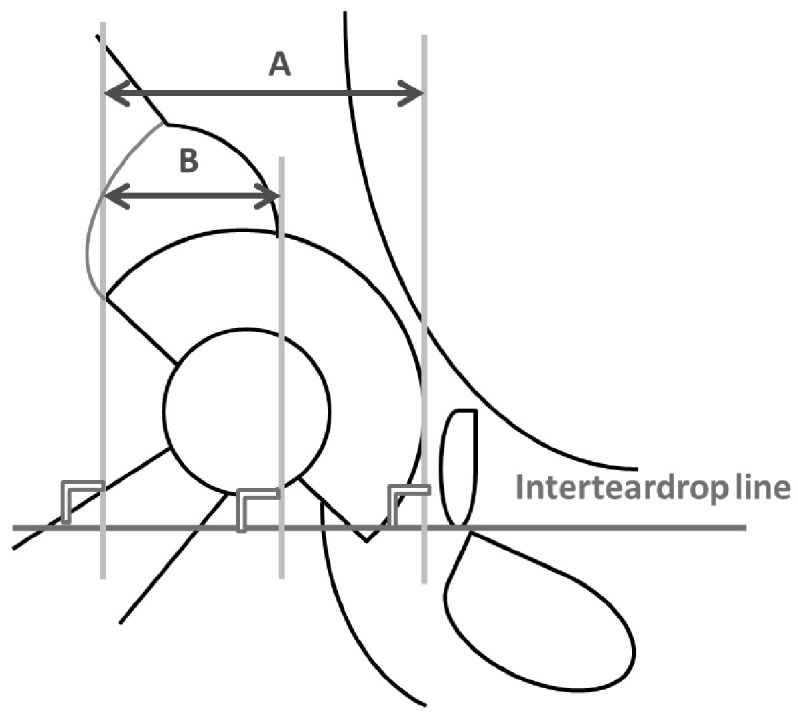
The proportion of the uncovered portion in relation to the entire metal implant surface (=horizontal distance of the uncovered portion (B)/horizontal distance between the medial and the lateral edge of the component (A)) × 100%).

**Figure 2:**
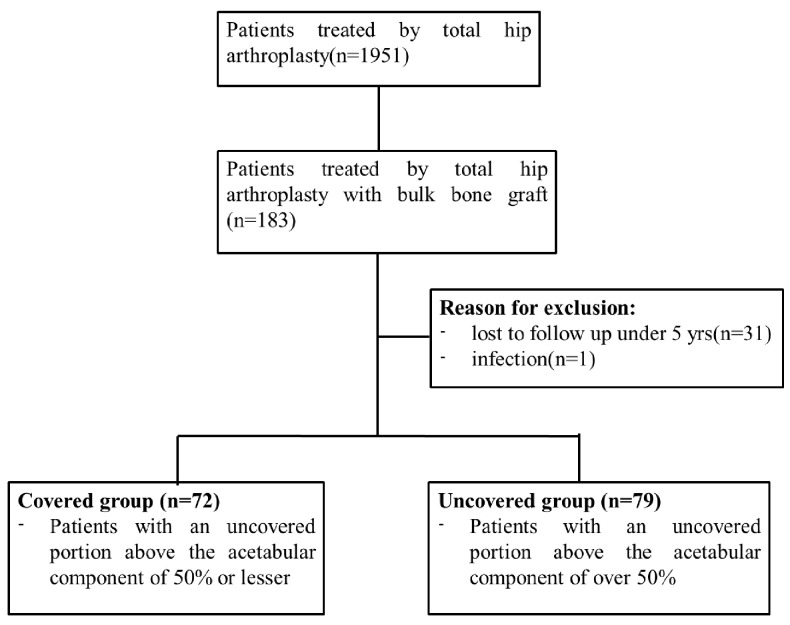
Patient selection flowchart.

They were divided into two groups based on the proportion of the acetabular component that was uncovered relative to the host bone, not the BBG. The proportion of the uncovered portion in relation to the entire metal implant surface was calculated using the following formula: (horizontal distance of the uncovered portion (B)/horizontal distance between the medial and the lateral edge of the component (A)) × 100% (Figure 1). Overall, 72 out of 151 patients had an uncovered portion above the acetabular component of 50% or lesser (covered group), while the remaining 79 patients had an uncovered portion of over 50% (uncovered group) (Figure 2).

**Figure 3:**
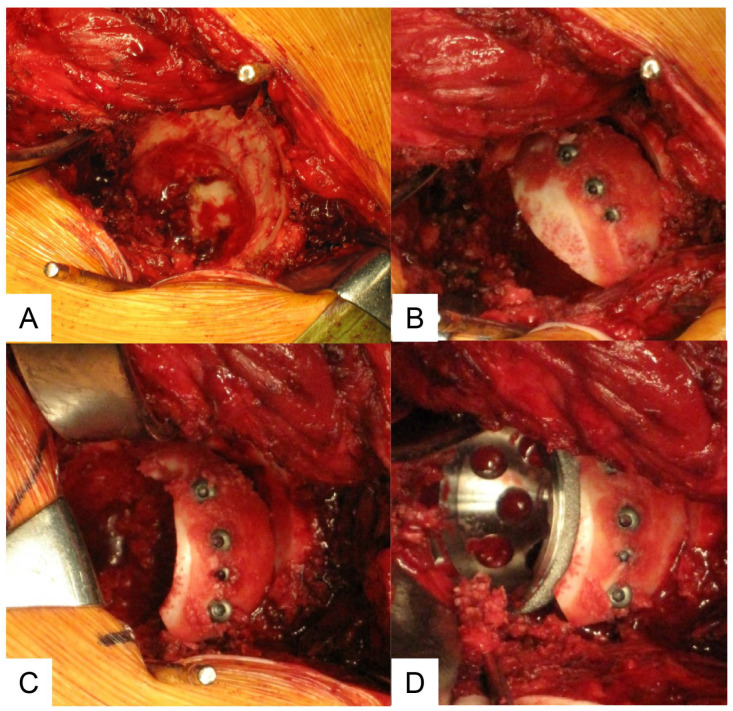
Surgical technique. (**A**) The lateral cancellous portion of the bulk bone graft is shaped with a bone saw to match the convexity of the pseudoacetabulum for accurate contact with the pseudoacetabular floor. (**B**) Fixation of graft bone. (**C**) Reaming again to fit the acetabular cup. (**D**) Fixation of the acetabular cup.

A posterolateral approach was used for all patients. In all patients, the cementless titanium porous acetabular component was fixed to the pelvis with or without screws. Superior segmental deficiencies of the acetabular bone were augmented with bulk autograft prepared from the ipsilateral femoral head. The grafting was accomplished in five steps. (1) The pseudoacetabular floor was reamed to expose cancellous bone and to facilitate invasion of fibrovascular repair tissue from the iliac bone marrow into the grafted bone. (2) The lateral cancellous portion of the bulk bone graft was shaped with a bone saw to match the convexity of the pseudoacetabulum for accurate contact to the pseudoacetabular floor (Figure 3A). (3) The cancellous surface of the graft was contacted in the prepared bed. The graft was fixed securely to the host with screws penetrating the outer walls of the iliac bone to prevent micromotion between the graft and host bone and to facilitate growth of repairing connective tissue into the graft. For fixation of graft bone, 3.5 mm canulated titanium screws (Stryker Orthopaedics, Mahwah, NJ, USA) were used in all patients (Figure 3B). (4) The reconstructed acetabular floor was reamed again to shape the bulk bone graft fixed to the pseudoacetabular floor (Figure 3C). (5) The acetabular component was implanted with several screws or without screws when firmly fixed (Figure 3D). The minimum follow-up was 5 years (mean 9.4 years; range, 5.0–13.2). Prior approval for the study was obtained from the Institutional Review Board. Four hips in the severe uncovered group and four in the covered group had been treated previously with one or more surgical procedures. The interval of non-weight bearing or partial weight bearing was determined by the surgeon and varied from one day to several weeks postoperatively. 

**Figure 4:**
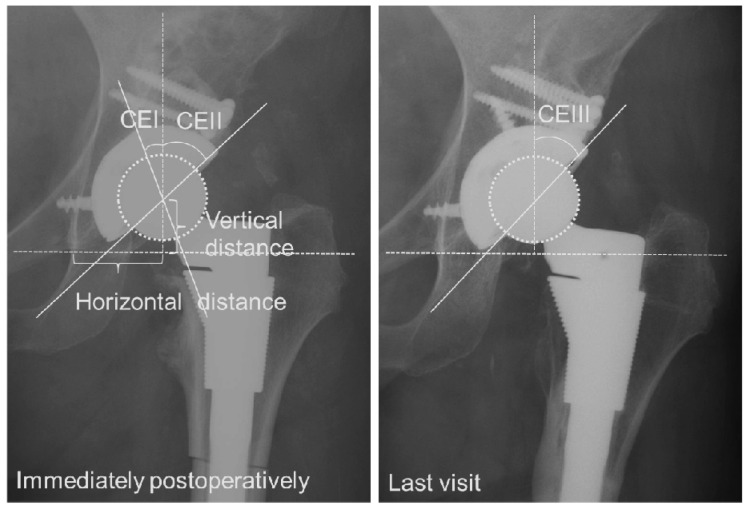
Measurements of representative cases. Three center edge (CE) angles were calculated: CE I, the angle between the vertical line of the femoral head center and the original lateral edge of the acetabulum (which was also the medial edge of the graft bone) immediately postoperatively; CE II, the angle between the vertical line of the head center and the lateral edge of the graft bone immediately postoperatively; and CE III, the angle between the vertical line of the femoral head center and the lateral edge of the graft bone at the last visit.

Standard anteroposterior (AP) radiographs were evaluated before operation, immediately after operation, and at final follow-up. The percentage of horizontal coverage by host and graft bone was determined on postoperative radiographs [25]. As a measure of coverage, three center edge (CE) angles were calculated: (1) CE I, the angle between the vertical line of the femoral head center and the original lateral edge of the acetabulum (which was also the medial edge of the graft bone) immediately postoperatively; CE II, the angle between the vertical line of the head center and the lateral edge of the graft bone immediately postoperatively; and CE III, the angle between the vertical line of the femoral head center and the lateral edge of the graft bone at the last visit. Radiographic findings of bone grafts at final follow-up were classified into three types: residual bone graft shadow, partial sclerosis, or complete incorporation [17] (Figure 4). Vertical and horizontal positions of the acetabular component were determined according to the methods described by Russotti [6]. A high hip center was defined as one with the center greater than 35 mm proximal to the inter-teardrop line.

Radiolucent lines at the acetabular bone–implant interface were recorded as described according to the method of DeLee and Charnley [26]. The inclination angle of the cup was determined as the angle subtended by the face of the cup and the inter-teardrop line on AP radiographs. Acetabular components that demonstrated a change in position of 4 degrees or more or migration of 4 mm or more were considered unstable [6,27]. For patients who subsequently underwent revision during follow-up, radiographic data from just before revision surgeries were used for analysis. NEOVISTA I-PACS version 1.09 R03 software (KONICA MINOLTA Inc., Tokyo, Japan) was used for all analyses.

The Student’s *t*-test and the Mann–Whitney U test were used for continuous variables. Pearson’s chi-squared test was used for dichotomous variables. Baseline characteristics were expressed as mean (standard deviation). A value of *p* < 0.05 was considered statistically significant. Data were statistically analyzed using R2.8.1 for Windows. Kaplan–Meier survival analysis was used to calculate the probability of retention of the original prosthesis with 95% confidence intervals. The intra- and inter-observer variability values in the CE angles between two orthopedic surgeons who differed from the primary surgeon were 0.921 and 0.843, respectively.

## 3. Results

**Figure 5:**
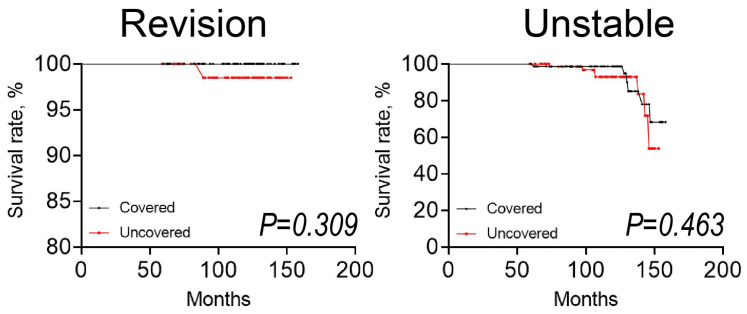
Kaplan–Meier survivorship analysis over five years. Survivorship curve for a bulk bone graft with the endpoint of revision for any cause and unstable fixation. The covered group is represented by the black dotted line and the uncovered group is represented by the red dotted line. The dots on the line indicate censored data.

The demographic features and average ages of the studied cases were relatively similar in the groups, while BMI (*p* < 0.001) and the proportion of male (*p* = 0.024) patients were higher in the uncovered group than in the covered group (Table 1). The distribution of Crowe type was statistically significantly different between groups. The Kaplan–Meier analysis revealed that the survival rate for the requirement of total hip arthroplasty revision was 98% (95% confidence interval, 94–100%) in the uncovered group compared to 100% (95% confidence interval, 100%) in the covered group at 10 years (Figure 5). The Kaplan–Meier analysis revealed that the survival rate for the radiographic unstable was 93% (95% confidence interval, 81–97%) in the uncovered group compared to 99% (95% confidence interval, 90–100%) in the covered group at 10 years. The differences in the survival rate between the uncovered and covered groups were not statistically significant (revision; *p* = 0.309, unstable; *p* = 0.463). Although no patients had acetabular component loosening attributable to collapse of the autograft in both groups, one hip was revised during follow-up. In this case, the hip prosthesis was revised at 7.5 years after the index procedure owing to breakage of a constrained cup, and the cup was firmly fixed to the acetabulum at the time of revision surgery. 

The mean cup diameters in the uncovered group, noted as 49.6 mm (42 to 58), were significantly larger (*p* < 0.001) than those in the covered group, noted as 47.7 mm (40 to 56). On the other hand, the cup inclination in the uncovered group of 40° (19 to 53°) was significantly smaller than (*p* = 0.062) that noted in the covered group of 42.4° (24 to 63°). The mean number of the screw for the component was 2.71 in the uncovered and 2.47 in the covered group (*p* = 0.131). The mean height of the hip center was 22.3 mm (8 to 36) in the uncovered group and 22.7 mm (9 to 39) in the covered group (*p* = 0.678). A high hip center was noted in one hip (1.3%) in the uncovered group and two hips (2.8%) in the covered group. The mean horizontal hip center position from the inter-teardrop line was 32.4 mm (23 to 43) in the uncovered dysplasia group and 31.4 mm (17 to 44) in the covered group (*p*= 0.445) (Table 2). The mean percentage of horizontal coverage of the acetabular component by graft bone was 58% (50.1 to 74.3) in the uncovered group and 39% (6.8 to 50.0) in the covered group. The mean CE improved from −14.2° (CE I; −39.3 to 12.8) to 46.9° (CE II; 19.6 to 67.9) immediately after surgery in the uncovered group and from −0.3° (CE I; −19.3 to 28.8) to 50.7° (CE II; 32.8 to 78.0) in the covered group (Table 3).

At final follow-up, 55 of the 72 hips (76.4%) in the covered group and 46 of the 79 hips (58.2%) in the uncovered group were completely incorporated; 7 (10.0%) in the covered group and 15 (19.0%) in the uncovered group had residual bone graft shadow; and 10 (13.9%) in the covered group and 18 (22.8%) in the uncovered group had partial sclerosis. Overall, 58 of the 72 cups (80.6%) in the covered group and 64 of the 79 cups (81%) in the uncovered group were considered to be stable, and 7 of the 79 cups (8.9%) in the uncovered group and 6 of the 72 cups (8.3%) in the covered group were considered to be unstable. However, in all unstable cases, the installation angle of the cup was displaced 6 months after the operation and was not moved until the last visit. In the covered group, 10 of the 72 cups (13.9%) had a radiolucent line under 2 mm thick around the acetabular component. In the uncovered group, 8 of the 79 cups (10.1%) had a radiolucent line (Table 4). 

## 4. Discussion

Despite numerous studies reporting the use of a bulk bone graft for the acetabular component, there is no consensus in orthopedic communities on the acceptable portion for graft bone coverage of the cementless acetabular component. In the present study, there was no significant difference in the survival rate using bulk bone grafts between patients with an uncovered portion of ≥50% and those with an uncovered portion of <50%. There was no loosening with the acetabular component placed at the true acetabulum even with an uncovered portion of ≥50%. Poor outcomes of bulk bone graft with a cement cup have frequently been reported [12,28,29]. For instance, Mulroy and colleagues have reported that grafts covering less than 40% tend to collapse, and they noted a failure of 46% of their cemented series by 11.8 years. In that report, they recommended at least 70% coverage of the acetabular component by the host bone to give stability and allow for adequate ingrowth on the bone [29]. In a related report, Rodriguez and colleagues recommended that coverage by the graft should be limited to <40% of the surface of the acetabular component for autogenous femoral-head bone-grafting with a cement cup [11]. They considered that as the graft covers the majority of the acetabular component, the potential for fatigue fracture of the graft and socket subsidence may increase.

On the other hand, good outcomes of bulk bone graft with a cementless cup have been documented in numerous studies with survival rate ranging between 91–97% over a period of 10 years [18]. However, uncovered portion above the cementless acetabular component was recommended not to exceed 40% to obtain sufficiently long-term fixation [12,23]. Li and colleagues reported that 30–50 coverage above cementless acetabular components is acceptable in the presence of initial implant stability to ensure a favorable setting for bone graft integration [30]. The acceptable amount of graft bone coverage of the cementless acetabular component necessary to ensure secure fixation has been controversial. 

Generally, the uncovered portion above the acetabular component with the host bone often exceeds 50% when setting the acetabular component on the true acetabulum. This study suggests that bulk bone grafting may be acceptable when the area not covered by host bone is greater than 50% to approximately 70%. As shown in Table 3, although the cup CE angle (CE II and III) was statistically smaller in the uncovered group compared to the covered group, an average of 45 degrees (larger cup inclination) was obtained at the final follow-up, indicating that the coverage was sufficient. It is possible to obtain firm fixation of a cementless acetabular component by means of bulk bone autograft, which provides additional anterolateral support for the cup in the face of host bone deficiency. The use of a cementless acetabular component provides lasting fixation for long time because the mechanical load on graft bone decreases when the host bone grows into the acetabular component. However, the cutoff value of the cup CE angle that predicts the prognosis could not be determined in this study and should be verified in future research. Favorable initial stability of the acetabular component is achieved by adhering to the superior, anterior, and posterior walls of the acetabulum. Table 2 shows that the mean cup diameter was smaller in the uncovered group, likely due to the higher number of Crowe type IV cases with hypoplastic acetabulum in the covered group.

The major limitations of this study include the fact that clinical result scores were not evaluated and that the follow-up periods of the studied cases were relatively short. However, in some cases, clinical scores are not sufficient to determine success of surgery. Indeed, Hooten and colleagues documented a limitation in bony union after structural bulk allografts for acetabular reconstruction although patients had good clinical scores and the radiographs showed incorporation between the graft and the host bone. In that case, allograft revascularization and remodeling were minimal even after 48 months [31]. Although good long-term results of THA with BBG performed with a similar surgical technique have been reported in recent years [15,16], longer follow-up is necessary to reach a conclusive decision in terms of bulk bone graft safety in hips with severe dysplasia. Second, there was no uniform duration of non-weight bearing after operation, and only standard AP radiographs, which reflect two-dimensional images of the hips, were used. Generally, three-dimensional images for bone defects in the acetabulum offer more accurate status of bone after implantation of the acetabular component. Third, the follow-up ratio in this study might be relatively low (82.5%), affecting the results.

## 5. Conclusions

Although the cutoff value for the uncovered portion could not be clarified in this study, the mid-term results for 50% to approximately 70% uncovered were comparable to those for 50% or lesser, which have previously been expected to perform well. Recently, biomechanically advantageous bone grafting techniques have been identified, and based on the results of this study, it may be possible to expand the indications for THA with bone grafting for developmental dysplasia of the hip.

## Figures and Tables

**Table 1:** Patient demographic characteristics.

	Covered	Uncovered	*p*-Value
Number	72	79	
Age, years	56 (8.0)	58 (8.8)	0.118
BMI, kg/m^2^	22.4 (3.0)	24.3 (3.7)	<0.001
Sex, n (%)			0.024
male	4 (5.6)	14 (17.7)	
female	68 (94.4)	65 (82.3)	
Crowe type, n (%)			<0.001
I	10 (13.9)	16 (20.3)	
II	15 (20.9)	33 (41.7)	
III	24 (33.3)	16 (20.3)	
IV	23 (31.9)	14 (17.7)	

Data are presented as mean (standard deviation).

**Table 2:** Cup size and position immediately after operation.

	Covered	Uncovered	*p*-Value
Median cup diameter, mm	47.7	49.7	<0.001
	(3.2; 40 to 56)	(2.9; 42 to 58)	
Cup inclination, degrees	42.3	40.0	0.062
	(8.2; 24 to 63)	(7.2; 24 to 68)	
Hip center, mm			
Vertical	22.7	22.3	0.678
	(6.2; 9 to 44)	(5.7; 8 to 21)	
Horizontal	31.4	32.5	0.445
	(5.1; 17 to 41)	(4.0; 23 to 57)	

Data are presented as mean (standard deviation; range).

**Table 3:** Graft coverage.

	Covered	Uncovered	*p*-Value
Graft coverage			
Horizontal coverage, %	39.4	57.9	<0.001
	(10, 6.8 to 49.9)	(5.5; 50 to 72)	
Center edge angle, degree			
CE I	−0.3	−14.2	<0.001
	(9.8; −19.8 to 28.8)	(8.5; −35.8 to 0.6)	
CE II	50.7	46.9	0.018
	(9.4; 34.4 to 74.8)	(9.7; 19.6 to 74.8)	
CE III	49.5	45.6	0.019
	(10.0; 31.0 to 81.1)	(8.8; 30.8 to 71.6)	

Data are presented as mean (standard deviation; range).

**Table 4:** Findings of graft bone and cup fixation at final follow up.

	Covered	Uncovered	*p*-Value
Findings of bone graft (radiolucent), n (%)			0.058
residual bone graft shadow	7 (9.7)	15 (19.0)	
partial sclerosis	10 (13.9)	18 (22.8)	
complete incorporation	55 (76.4)	46 (58.2)	
Cup fixation, n (%)			0.774
stable	56 (77.8)	64 (81.0)	
radioluscent line (<2 mm)	10 (13.9)	8 (10.1)	
unstable	6 (8.3)	7 (8.9)	

Data are presented as mean (standard deviation; range).

## Data Availability

The data presented in this study are available upon request from the corresponding author.
